# ‘It’s Really About Wellbeing’: a Canadian Investigation of Harm Reduction as a Bridge Between Mental Health and Addiction Recovery

**DOI:** 10.1007/s11469-020-00239-7

**Published:** 2020-02-20

**Authors:** Mary Bartram

**Affiliations:** Faculty of Law and the Institute for Health and Social Policy, McGill University, Charles Meredith House, 1130 Pine Avenue West, Montreal, Quebec H3A 1A3 Canada

**Keywords:** Mental health, Addiction, Recovery, Harm reduction, Canada, Wellbeing

## Abstract

Recovery is a key concept driving system transformation in both the addiction and mental health sectors, with shared roots in advocacy and a shared focus on hope in the face of stigma, self-determination and meaningful lives. Nevertheless, while mental health recovery is possible even with on-going symptoms, addiction recovery generally starts with or leads to abstinence. This disconnection undermines coherence at the policy level and exacerbates fragmentation between services and supports in the mental health and addiction sectors in Canada and internationally. At the same time, harm reduction, which does not require abstinence, has been gaining ground in the Canadian addiction sector. This qualitative policy study explores the potential for harm reduction to bridge the gap between mental health recovery and addiction recovery in the Canadian context, drawing on diverse experiences from the mental health and addiction sectors. The findings could be adapted  internationally to address similar policy challenges.

International observers with a keen interest in mental health and addiction policy may find themselves perplexed by the existence of parallel but seemingly independent recovery movements in Canada. Within a year of each other, the Mental Health Commission of Canada (MHCC) and the Canadian Centre for Substance Use and Addiction (CCSA) released independent national statements setting out a vision for mental health recovery on the one hand and addiction recovery on the other (MHCC 2015; CCSA 2017, p. 25). These statements illustrate that recovery is a key concept, driving system transformation in both the addiction and mental health sectors, with shared roots in advocacy and a shared focus on hope in the face of stigma, self-determination and meaningful lives. Nevertheless, they also illustrate a core conceptual disconnection: while mental health recovery is possible even with on-going symptoms, addiction recovery generally starts with or leads to abstinence. This disconnection undermines coherence at the policy level and exacerbates fragmentation between services and supports in the mental health and addiction sectors, which is particularly problematic given shared risk and protective factors and high rates of concurrent mental health and substance use disorders. At the same time, harm reduction, which does not require abstinence, has been gaining ground in the Canadian addiction sector in response to both an opioid crisis and a shift to the left at the federal level. Through interviews and focus groups with diverse stakeholders in the mental health and addiction sectors, this qualitative policy study explores the potential for harm reduction to bridge the gap between mental health recovery and addiction recovery in the Canadian context.

Similar tensions regarding recovery and harm reduction are playing out in comparable countries. In the UK, there is a disconnection between mental health–focused recovery colleges and the emergence of recovery capital as a core concept in the addiction sector (Best and Laudet 2010; Whitley et al. 2019). In Australia, the National Framework for Recovery-Oriented Mental Health Services focuses almost exclusively on mental health recovery (Australian Health Ministers’ Advisory Council 2013). In the USA, the Substance Abuse and Mental Health Services Administration (2012) has developed an integrated definition of mental health and addiction recovery, but integrated approaches to policy and service delivery are challenged by separate mental health and addiction structures and funding streams. In all three of these countries, debates of varying intensity regarding harm reduction are on-going (Des Jarlais 2017; Vumbaca 2018; Winstock et al. 2017).

The similarities and tensions between mental health recovery, addiction recovery and harm reduction can be seen through a closer comparison of definitions. Anthony (1993) drew on various lived experience accounts to develop the mental health sector’s foremost definition of recovery, as “a way of living a satisfying, hopeful, and contributing life even with limitations caused by illness” (Anthony 1993, p. 15). While this definition emphasizes how cure is not necessary for recovery, definitions of recovery in the addiction sector have tended to emphasize abstinence as a necessary starting point. Recovery and sobriety have been synonymous in the Alcoholics Anonymous movement for over 80 years. Although the focus on abstinence has been softening under the influence of harm reduction, current definitions of recovery in the addiction sector continue to invoke abstinence. For example, between 2015 and 2017, the definition of recovery used by CCSA shifted from “a process of personal growth along a continuum leading to abstinence” (CCSA 2017, p. 25) to “in addition to abstinence or stopping uncontrolled substance use, recovery implies improved health, function, and quality of life” (McQuaid 2017, p. 14). The Substance Abuse and Mental Health Services Administration’s integrated definition also simultaneously introduces flexibility while invoking abstinence: “Overcoming or managing one’s disease(s) or symptoms—for example, abstaining … if one has an addiction problem—and for everyone in recovery, making informed, healthy choices that support physical and emotional well-being” (2012, p. 3).

By contrast, definitions of harm reduction in the addiction sector very explicitly exclude the necessity of abstinence. For example, in a recent consultation document for a renewed substance use strategy, Canada and  Health Canada (2018) clearly state that “harm reduction aims to reduce the negative health, social and economic impacts of substance use on individuals, their families and communities, without requiring abstinence” (p. 17). Applications range from safer use guidelines for legal substances such as alcohol and (as of 2018) cannabis, to supervised consumption sites, needle exchange programs and opioid agonist therapy (methadone or suboxone treatment) designed to reduce harms associated with illegal drugs such as heroin. Harm reduction has much in common with both mental health recovery and addiction recovery, including origins in a grass roots movement and shared principles regarding the importance of self-determination and the voices of people with lived experience (Harm Reduction Coalition n.d.). However, harm reduction’s defining feature and the one that sets it apart from addictions recovery is its non-judgmental stance regarding substance use, something which is conceptually similar to mental health recovery’s acceptance of ongoing symptoms.

While the conceptual disconnections between addiction recovery and mental health recovery and between addiction recovery and harm reduction are well-known issues in the field, they are rarely discussed in academic or grey literature. One prominent exception comes from Davidson and White (2007), who argue that the differences between mental health and addiction recovery can be overcome by focusing on the considerable common interest in hope, empowerment and recovery of meaningful lives in community. “With this shared foundation in place, differences that have existed historically between the recovery visions of the mental health and addictions systems could then provide opportunities for synergistic growth in both” (p. 114). These efforts are being helped by a new interest in reaching beyond abstinence on the part of the addiction recovery movement. For example, recovery capital refers to the internal and external resources that support and sustain recovery (Best and Laudet 2010). At the same time, recovery capital does not explicitly address the conceptual disconnection between harm reduction and addiction recovery. Reaching beyond abstinence is not the same thing as not requiring abstinence, but rather widens the definition of recovery to include the factors that facilitate and sustain abstinence. More recently, a new USA-based initiative is focused squarely on reducing tension between addiction recovery and harm reduction by taking aim at media depictions of harm reduction and abstinence as “warring camps” (Changing the Narrative 2019).

Theory regarding the relationship between ideas, institutions and interests in shaping public policy suggests that a coherent vision is particularly important when challenging entrenched policy paradigms (Gauvin 2014; Hall 1993). A coherent vision provides an anchor for policymakers to hold on to in the face of resistance to change from existing institutions and interests. Certainly, the vision of a recovery-oriented mental health system has been challenging an entrenched biomedical paradigm in Canada and internationally (Mulvale and Bartram 2009, 2015; Piat and Sabetti 2012) and is increasingly playing a similar role in addiction system transformation (CCSA 2017; Kelly and Hoeppner 2015). Nevertheless, these efforts have been hampered by entrenched divisions between the mental health and addiction sectors, and between abstinence-oriented addiction recovery and harm reduction. While debates about concepts may seem removed from the front line where fragmentation between mental health and addiction services has real impacts on people’s lives, conceptual coherence does have the potential to make a real difference.

A unified vision to guide reforms in the mental health and addictions sectors is particularly relevant in the current Canadian policy context. While access to services is only one part of a recovery-oriented system, improving access to both mental health and addiction services is a shared priority across federal, provincial and territorial governments, and is being supported by a targeted federal transfer of $5 billion through to 2027 (Bartram 2017). At the provincial and territorial policy level, mental health and addiction strategies that are both integrated and recovery-oriented have become the norm (Newfoundland and Labrador 2017; Virgo 2018). At the service system level, integration of mental health and addiction services continues to be a challenge (Addiction and Mental Health Collaborative Project Steering Committee 2015). Meanwhile, governments are grappling with an overdose crisis that is compounded by politically driven shifts in harm reduction policies (Kerr et al. 2017). All of these developments have opened a window of opportunity for a coherent vision—one which unifies mental health recovery, addiction recovery and harm reduction—to have a meaningful impact on public policy.

This study is part of a broader research project which aims to generate dialogue and knowledge regarding the relationship between mental health recovery, addiction recovery and harm reduction. A complementary concept analysis (Bartram 2019) integrates mental health, mental illness, harm and substance use into a unified conceptual framework (see Fig. 1). Within this framework, movement toward recovery and wellbeing can occur along any of the four continua with common influences from individual, social and structure factors. By talking to people who are immersed in these relationships and conceptual issues, this qualitative policy study aims to get a richer understanding of the potential for harm reduction to bridge the conceptual disconnection between recovery in the mental health and addiction sectors.

**Fig. 1:**
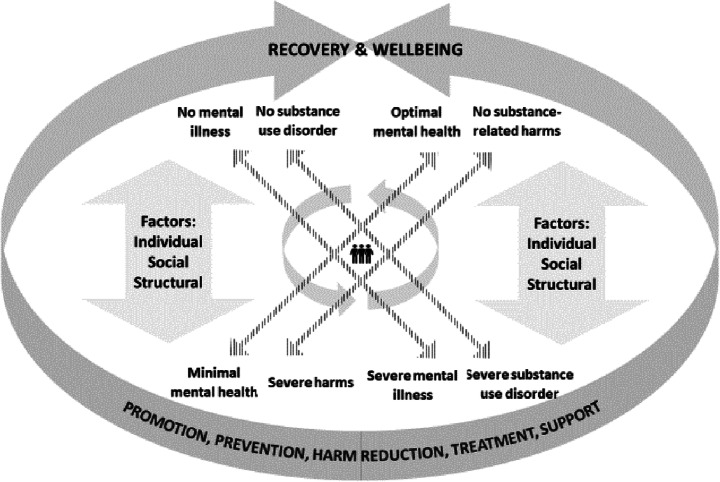
Integrated model of mental health and addiction recovery and well-being (Courtesy of Bartram 2019)

## Methods

Using a phenomenological approach, interviews and focus groups were conducted with people with diverse experiences of the relationship between recovery and harm reduction (Starks and Brown Trinidad 2007). By means of an iterative purposive sampling process, 21 participants were recruited with overlapping experiences of service delivery, living with mental health and/or substance use problems, advocacy, policy and research. In keeping with the need to focus on both addiction recovery and harm reduction, just over half (12) of the participants had a primarily affiliation with the addiction sector, six participants had a primary affiliation with the mental health sector and three were firmly affiliated across both sectors. Participants were all working adults of varying ages over 24 and living in North America (primarily Canada) with four of ten Canadian provinces represented. Ethics approval was obtained from McGill University and informed consent was obtained from each participant.

The author conducted semi-structure interviews and focus groups by phone, videoconferencing or in-person in April and May 2019 (the guiding questions can be found in Table 1). Interviews were transcribed and coded by the author using NVivo 12. To maintain confidentiality, participants are only identified by research participation number (from RP1 to RP21). The initial round of coding identified 38 nodes and 17 subnodes, which were subsequently distilled down to the three overarching themes discussed below.

**Table 1:** Guiding questions for interviews and focus groups

What are the similarities and differences between mental health recovery and addiction recovery? What is the relationship between harm reduction and addiction recovery? In what ways could harm reduction strengthen or weaken the relationship between mental health recovery and addiction recovery? What are the opportunities and challenges for harm reduction to act as a bridge between the mental health and addiction sectors? What would the implications of such as bridge be for policy? For system transformation? For service delivery? For people living with mental health and/or substance use problems? What other approaches could be used to foster a shared vision of recovery in the mental health and addiction sectors?	

The author has considerable experience in the mental health and addiction sectors that was both an asset and liability during the study. These experiences helped the author to join with participants and to deepen the conversations, but also meant that the author’s preconceived beliefs could influence the interviews and subsequent analysis. To guard against this risk, the findings were validated and refined through consultations with additional experts in the addiction, mental health and public policy sectors. These consultations included feedback at two academic conferences, four reviews of the draft manuscript and one validation session with a recovery research team.

## Results

The three overarching themes identified by the author include (1) competing views on whether the benefits of integration outweigh the risks, (2) growing common ground centred around meeting people where they are at while offering support for a better life and (3) wellbeing as a more promising bridge than harm reduction. These themes are summarized in Table 2.

**Table 2:** Summary of key themes

Competing views on integration	
Benefits	- Explicit value of an integrated vision “to get the kind of meaningful, deep change that you need”
	- Importance of leadership from people with lived experience “to be at the foundation of this conversation, at the front of this debate”
	- Need for mental health and addiction system integration given “the linkage between the two”
	- Recognition of the social determinants of health “as a large contributing factor to both mental health and substance abuse”
Risks	- Distinct identities can get lost “you are going to lose addiction”, “there is a danger in introducing harm reduction language into mental health”
	- Concepts are already controversial enough on their own, from harm reduction’s “ability to really polarize people” to the “backlash” against addiction recovery to “the risks involved” in mental health recovery
Meeting people where they are at while offering support for a better life	
Meeting where at	- Meeting people where they are at with compassion and lack of judgement “we are going to continue to support you where you are at in that moment”
	- Recognition of relapse as a common occurrence “it’s not what we wish for people, but it is likely”
	- Harm reduction on a continuum toward long-term recovery “because if people die, they cannot recover”
Offering support	- Respect right to self-determination but at least offer support, education and the belief that “I could have a good life”
Wellbeing as a more promising bridge than harm reduction	
	- Whether harm reduction, mental health recovery or addiction recovery “the final goal is very much to achieve wellness at the end of the day”
	- Adopt a more expansive and holistic perspective “is the common bridging of recovery a necessary component of wellness?”

### Competing Views on Whether the Benefits of Integration Outweigh the Risks

Participants were divided over on whether the benefits of an integrated approach to mental health recovery, addiction recovery and harm reduction outweigh the risks. Some participants were explicit in seeing the value of an integrated vision:

If you have a system where some people think harm reduction is … evil, and that if you are not doing abstinence-based programming you are not really in recovery, and some people think that it’s ok-- if you do not build consensus around that vision and have conceptual clarity, it is really hard to get the kind of meaningful, deep change that you need. Once we started the work, those issues did not come up because we had a shared agreement and a shared vision that we had bought into moving forward. (RP6)

There have certainly been some significant challenges in the competing paradigms of mental illness and addictions that have caused tremendous grief. (RP20)

I think that is really important to bring to the dialogue, from whatever perspective you are coming from or entrenched in, if you can keep the longer goal and the higher vision of hope for people it brings us to a common point. (RP14)

One takeaway I had was the importance of not letting the internal tension between groups within the field of substance use, I am thinking of harm reduction and recovery in particular, get in the way of the type of comprehensive service integration that needs to happen between mental health and addictions. (RP18)

Further, in keeping with shared recovery and harm reduction principles, participants stressed the importance of leadership from people with lived experience for the development of a shared vision.

You guys aren’t working together and we insist that you do because we are the ones that suffer when you do not. And we are the ones that you get funded to help. And now we are visible and we are public and we are in front of your funders talking. Listen up. (RP7)

If we have learned anything from recovery it’s that people with lived experience need to be at the foundation of this conversation, at the front of this debate. (RP10)

With reference to how strongly mental health and substance use are interlinked, participants also focused more broadly on the need for integration between service systems.

It’s always making sure that there’s an awareness of the linkage between the two and that the addiction … is often the result of mental illness of some sort, or trauma, or those kinds of things. So if you are not dealing with one, often you are not actually going to have a positive result for the other. (RP8)

I think there’s just a lot of things that both sectors can teach each other, and I think that that would foster shared vision by … acknowledging that there’s a significant shared population across mental health and addictions, that these are not distinct populations. And I think that that’s incredibly important. (RP2)

The majority of the people who have surely the most significant mental health challenges also have substance use challenges, so in my mind it does not make sense to try to tease out whether this person has a mental illness and therefore we should have a different set of strategies. (RP6)

Similarly, participants pointed to benefits of integration for joint action on social determinants of health.

When people have mental health and/or addictions issues … their other social determinants of health are impacted, whether it be their income, their housing, their employment status, or their education status. So it’s that kind of spiral impact. (RP1)

I think there is more recognition, perhaps growing in the substance use area, that it’s a community-based approach, and peer support, and recognizing the determinants of health as a large contributing factor to both mental health and substance abuse. (RP19)

Mental health recovery is really in many respects good mental health promotion, and then addiction recovery … has often historically been about ending the relationship to the source of harm, although I see that as a symptom of something else. Which leads to overlapping convergences in terms of action on protective factors, things around social connectivity, a job, healing underlying trauma or addressing social determinants of health. (RP20)

In contrast to this broad support for increased integration, participants also pointed to significant risks. For example, they spoke to how each of the mental health, addiction and harm reduction sectors are protective of their distinct identities. This sentiment came through most strongly with regard to the addiction sector.

Sometimes addiction and drug use fall under the mental health umbrella …. But drug use, substance use disorders get kind of lost in that so maybe that distinction needs to be better made to better tailor services. (RP15)

Addiction comes with its own set of stigma, its own set of everything. There’s lots of concern when you do merge that what’s going to happen is you are going to lose addiction. So my gut would tell me, “not a good thing.” (RP21)

While less pronounced, harm reduction and mental health perspectives were also wary of integration.

I still think [recovery’s] a very white concept. …The addictions recovery movement has assumptions about recovery, the harm reduction movement is questioning every possible assumption that’s out there, right? I would think those two are not even comparable as movements. If we are talking about recovery from addiction there is a lot of attention to trauma, but if you are recovering from poverty how do you do that? You’re recovering from-- this does not even make sense, right? That’s not an individual’s choice over that. (RP21)

[T]here might be a bit of a danger in introducing [harm-reduction] language into mental health … where it brings up perhaps a focus on eliminating symptoms, ensuring treatment adherence, things like that, which are not really compatible with our philosophy of recovery. (RP12)

This idea that people have to hit a wall, they have to lose everything … before they recognize that the addiction is bigger than them. I hate that. I hate it. (RP20)

Participants also pointed to the downsides of integrating concepts that are already controversial enough on their own.

It’s not as if there is not a massive evidence base to support harm reduction, but it somehow has this ability to really polarize people. I think its rooted in stigma and illegality around addiction and the use of substances. (RP10)

As we talk about recovery, I think there’s quite a backlash against just that because who or is anyone to tell me that I cannot live using? Right? …Whose right is it to ask somebody to not use if they are going to feel all these feelings or be re-traumatized? I just think there’s all these conversations that always tend to kind of come up around talking about recovery. (RP21)

There is an emphasis on choice and self-determination in mental health recovery. And we know that the risk involved is one of the biggest barriers for the psychiatric community to embrace recovery in its fullness. (RP2)

### Meeting People Where They Are at While Offering Support for a Better Life

Despite being divided on the benefits of integration, participants’ responses suggest an emerging consensus. Whether participants were speaking from a mental health, addiction or harm reduction perspective, the most consistent message in the study as a whole was the importance of meeting people where they are at while also offering support for a better life. Meeting people where they are at with compassion and lack of judgement shone through many of the interviews.

We’re not going to kick you out of a program. We’re not going to judge for your use. We’re going to continue to support you where you are at in that moment. (RP3)

[T]he [peer support groups] I am familiar with … all have the same thing – abstinence is not required for membership. They are all harm reduction based. They are all based on compassion and acceptance. Someone may want to have a better life but not be capable of it. If you go there drunk or high for twenty years all that ever happens is they ask you if you are going to come back tomorrow… So there is that acceptance that you can be ill in our community and that you do not have to get better for us to care for you. That is the heart of the recovery process. (RP7)

We always felt that it was important to start where that person was and so the person who had the gambling problem who said … this is what I’m going to do, this I what I’m not going to do. …For some people it might make a whole lot of sense for them to be free from all substances… But if that wasn’t their choice, then that’s not their goal in their recovery. (RP1)

While some participants from the addiction sector continued to invoke abstinence, this ideal was tempered by a recognition of relapse as a common occurrence with substance use problems just as with mental health problems.

[W]e know that a very small proportion of [people who are trying to be abstinent] are going to just stop using and then never use again. So there is a place even with an abstinence-based definition of recovery for harm reduction because people will use occasionally. (RP21)

You’ve learned a lot in your recovery over the years. Your relapse has taught you something yet again. …It’s not what we wish for people, but it is likely. And depending on the substance that people are using, the likelihood is even greater. (RP1)

So we have got to tell them, yes it’s good to have complete abstinence but do not ever get too tied up with that because what happens is when you do slip you feel like you have failed and have let down all your friends. That’s not what its about. (RP8)

Mental health comes from a place of “I am living with.” “I am a person living with substance use disorder” … speaks to the chronic nature and the reoccurrences that can happen, … it speaks to a different message than the [addiction] recovery message which would mean that I am good now and I am going to be great forever and I am in abstinence and all of my problems are solved. (RP7)

Further, reducing use was perceived as an important step in the recovery journey. In this sense, harm reduction was situated by some participants along a continuum toward long-term recovery.

Harm reduction can be effective in progressing people along the [recovery] journey. ...If they have improved wellness or they have avoided some potential harms then it may lead to improvements in their quality of life. (RP15)

Long-term recovery is always our goal and harm reduction is the means to an end, it’s not the end. … We saw harm reduction on a continuum of ways that we help people, and a very necessary part of a truly recovery-oriented system because if people die, they cannot recover. (RP6)

If abstinence is required before treatment for different concurrent issues, then you might be denying someone that treatment because abstinence is too hard to achieve in the short term even if that is their goal. If we take it into the context of harm reduction …, then it could be a stepwise thing. You reduce a bit, you start treatment, you reduce a bit more. (RP16)

We even considered contemplation as part of that spectrum of harm reduction. So even if you have not actually taken active steps to reduce use or use safely…, just the thought of considering reducing use or changing use could be considered part of that. (RP14)

While participants were passionate about meeting people where they are at without judgement and with respect for people’s right to make their own decisions, they were also adamant about at least offering support for a better life, whether through education, information, role modelling, or simply conveying a message of hope and optimism.

When you treat people respecting where they have been and appreciate where it is that they are coming from and what it is they want to do, then that opens the door for them to maybe feel that, “Well, maybe I am worth doing something else.”(RP1)

At the methadone clinic in the city, its almost like a herd of cattle going to slaughter, in and out, in and out, there’s no talking about your addiction, there’s no talking about how your life is getting on. Education is the key. …This is what’s going on, this is why we are doing it. Have information so that if you are ever trying to get clean and sober, try to go here. (RP8)

I am not ever judging somebody if they are still using. But if they come in the room

and see a lot of people, especially somebody who used a year ago, all of a sudden they … say … if you can do it, I can do it. (RP8).

I need safety and other people telling their stories that I can hear. And an internal optimism that [has] the decency to not know what happens for me, the grace not to think you are going to save me, but that you believe I could have a good life. That I will probably have to learn some things and get some new tools but you are confident that it’s possible…. I do not want to see a bunch of people warehoused and capped off as in, we got them out of the emergency room, this is pretty good for them. (RP7)

### Wellbeing as a More Promising Bridge than Harm Reduction

While not quite as strong as the consensus around meeting people where they are at, many participants proposed the idea of wellbeing as a more promising bridge than harm reduction between mental health recovery and addiction recovery.

For addiction recovery, mental health recovery and harm reduction, the final goal is very much to achieve wellness at the end of the day. It’s very much impacted by external factors and also facilitated by different types of support networks. (RP17)

Harm reduction is deficit-based language. Using strength-based language around living well would maybe be a better bridge between the concepts of recovery in both fields. (RP12)

Some participants went further and proposed abandoning concerns over recovery in favour of a more expansive and holistic perspective.

I think mental health and addiction still live in those silos so much and as we are asking how can we bridge them together, is there another way of approaching that question? …Is there another way of looking at it that does not kind of dissect everything and then try to put it together into a new piece? (RP21)

So harm reduction, it’s really about wellbeing…. I would use the term wellbeing because of the stigma and misunderstanding of recovery. (RP7)

If the agreed goal or end state is wellness, is the common bridging of recovery a necessary component of that? (RP18)

We went through this conceptual evolution where we went from just getting people into communities, to people being a part of communities, to create[ing] healthy communities. …[H]ow do we help people stay well, how do we message things that people can do to stay psychologically healthy, how do we intervene much earlier before people have a diagnosis? (RP6)

## Discussion

The overarching themes around integration, meeting people where they are at and wellbeing identified in this qualitative study suggest that harm reduction is already bridging the disconnection between mental health recovery and addiction recovery in practice in this largely Canadian sample. Although support for integration was divided, many participants saw the benefits. The greatest area of consensus—meeting people where they are at while offering support for a better life—clearly illustrates the influence of harm reduction on abstinence-based approaches to recovery. Nevertheless, the wariness expressed regarding integration suggest that harm reduction may be too controversial and too distinct to be of much use in the development of a unifying vision, particularly are both mental health recovery and addiction recovery are similarly controversial and distinct. By integrating harm reduction, substance use, mental health, mental illness continua along with shared risk and protective factors into a unified model of recovery and well-being, the proposed conceptual framework (Bartram 2019, see Fig. 1) is consistent with the perspectives and experiences of the research participants. However, these perspectives and experiences strongly suggest that it may be more effective to move toward a shared vision by focusing on the bigger picture. Rather than using harm reduction as a bridge, a focus on wellbeing or meeting people where they are at could side-step controversies and conceptual quagmires, and allow the mental health recovery, addiction recovery and harm reduction movements to maintain a separate identity while exploring common ground.

Two key limitations of this phenomenological study are the reliance on a single researcher and the exclusion of youth perspectives. Phenomenological methods rely heavily on the ability of the research team to distil the experience and perspectives of participants into themes. To some extent, a single researcher can validate his or her understanding of a participant’s point of view through reflective listening during the interview and focus group process, and the author did validate the findings through consultation with additional experts. Nevertheless, a research team would have been able to strengthen the credibility and validity of the findings by counterchecking each other’s coding and analysis.

Phenomenological methods also rely on the author to determine when enough participants have been included to reach a sufficient level of understanding of a particular phenomenon. While 21 participants appeared to the author to be sufficient for achieving a general understanding of diverse perspectives on the relationship between harm reduction and recovery, the exclusion of youth under 25 is a key limitation. In another complementary study on harm reduction in post-secondary settings, youth with experiences in peer support and health promotion expressed a strong commitment to harm reduction as a unifying vision across a wide range of health and social issues, from safer partying to safer sex, from pedagogy to racism, from mental health problems to substance use problems and from sexual violence prevention to suicide prevention (Bartram et al. 2019). In keeping with broader discourse regarding safe spaces in post-secondary settings, a unifying theme across all of these domains is that harm reduction should only be approached “in a space of compassion and non-judgment” (University of Toronto 2019) with “zero tolerance for shame” (Bartram et al. 2019). It may be that as today’s youth take on leadership positions in society, they will elevate harm reduction to a unifying concept in new and different ways than have been seen to date.

The strengths of this study lie in the richness of the perspectives and experiences captured in the key themes and in its policy relevance. In Canada and internationally, an on-going overdose crisis and burgeoning recovery and harm reduction movements are galvanizing people in the sectors and the broader public. In the Canadian context, mental health and addiction are at the top of government health policy agendas, a 10-year targeted federal transfer is in place, and there are specific calls for increased integration. A window of opportunity has opened in Canada to shape a more unified vision for the mental health, addictions and harm reduction sectors, while respecting the distinct identity of each. The findings from this study suggest that a logical next step would be a Canadian wellbeing summit (as opposed to a recovery summit) to explore the possibility of such a unified vision, with engagement of people from diverse sectors and perspectives (including youth) and leadership from people with lived experience. Such a vision could be adapted internationally to address similar policy challenges.

## Conclusions

While parallel mental health recovery and addiction recovery movements are gaining strength in Canada and internationally and share considerable common ground, important conceptual differences regarding the necessity of cure and abstinence undermine coherence at the policy level and exacerbate fragmentation in services and supports. This qualitative policy study uses phenomenological methods to develop a rich understanding of diverse perspectives and experiences regarding the potential for harm reduction, which does not require abstinence, to act as a bridge between mental health recovery and addiction recovery. Overarching themes from interviews and focus groups with 21 participants include (1) competing views on whether the benefits of integration outweigh the risks, (2) growing common ground centred around meeting people where they are at while offering support for a better life and (3) wellbeing as a more promising bridge than harm reduction. While harm reduction is already bridging the disconnection between mental health recovery and addictions recovery in practice, it may be too controversial and too distinct to be of much use in the development of a unifying vision. At a time when mental health and addiction are top priorities for public policy in Canada, there is a window of opportunity to bring people from diverse experiences and ages together to develop a more unified vision of wellbeing for the mental health, addictions and harm reduction sectors, while respecting the distinct identity of each. In keeping with theories regarding the role of ideas in public policy, such a vision could pave the way for changes in institutions and services that have a direct impact on the population in Canada and beyond.
